# Large scale variation in *Enterococcus faecalis *illustrated by the genome analysis of strain OG1RF

**DOI:** 10.1186/gb-2008-9-7-r110

**Published:** 2008-07-08

**Authors:** Agathe Bourgogne, Danielle A Garsin, Xiang Qin, Kavindra V Singh, Jouko Sillanpaa, Shailaja Yerrapragada, Yan Ding, Shannon Dugan-Rocha, Christian Buhay, Hua Shen, Guan Chen, Gabrielle Williams, Donna Muzny, Arash Maadani, Kristina A Fox, Jason Gioia, Lei Chen, Yue Shang, Cesar A Arias, Sreedhar R Nallapareddy, Meng Zhao, Vittal P Prakash, Shahreen Chowdhury, Huaiyang Jiang, Richard A Gibbs, Barbara E Murray, Sarah K Highlander, George M Weinstock

**Affiliations:** 1 Division of Infectious Diseases, Department of Medicine, University of Texas Medical School, Houston, Texas 77030, USA; 2 Center for the Study of Emerging and Re-emerging Pathogens, University of Texas Medical School, Houston, Texas 77030, USA; 3 Department of Microbiology and Molecular Genetics, University of Texas Medical School, Houston, Texas 77030, USA; 4 Human Genome Sequencing Center, Baylor College of Medicine, Houston, Texas 77030, USA; 5 Department of Molecular and Human Genetics, Baylor College of Medicine, Houston, Texas 77030, USA; 6 Department of Molecular Virology and Microbiology, Baylor College of Medicine, Houston, Texas 77030, USA

## Abstract

**Background:**

*Enterococcus faecalis *has emerged as a major hospital pathogen. To explore its diversity, we sequenced *E. faecalis *strain OG1RF, which is commonly used for molecular manipulation and virulence studies.

**Results:**

The 2,739,625 base pair chromosome of OG1RF was found to contain approximately 232 kilobases unique to this strain compared to V583, the only publicly available sequenced strain. Almost no mobile genetic elements were found in OG1RF. The 64 areas of divergence were classified into three categories. First, OG1RF carries 39 unique regions, including 2 CRISPR loci and a new WxL locus. Second, we found nine replacements where a sequence specific to V583 was substituted by a sequence specific to OG1RF. For example, the *iol *operon of OG1RF replaces a possible prophage and the *vanB *transposon in V583. Finally, we found 16 regions that were present in V583 but missing from OG1RF, including the proposed pathogenicity island, several probable prophages, and the *cpsCDEFGHIJK *capsular polysaccharide operon. OG1RF was more rapidly but less frequently lethal than V583 in the mouse peritonitis model and considerably outcompeted V583 in a murine model of urinary tract infections.

**Conclusion:**

*E. faecalis *OG1RF carries a number of unique loci compared to V583, but the almost complete lack of mobile genetic elements demonstrates that this is not a defining feature of the species. Additionally, OG1RF's effects in experimental models suggest that mediators of virulence may be diverse between different *E. faecalis *strains and that virulence is not dependent on the presence of mobile genetic elements.

## Abstract 2

A comparison of two strains of the hospital pathogen *Enterococcus faecalis* suggests that mediators of virulence differ between strains and that virulence does not depend on mobile gene elements

## Background

Enterococci have emerged over the past few decades as the second to third most common cause of nosocomial infections, including urinary tract and soft tissue infections, bacteremia, and endocarditis [1-3]. They are well equipped to thrive in environments with heavy antibiotic usage due to both their intrinsic resistance to antibiotics and their talent for swapping genetic information, which allows them to gain and share resistance determinants. Entecococcal infections are predominantly caused by *E. faecalis *and *E. faecium*. However, many, if not most, strains of these species are harmless commensals, with some enterococci being marketed in Europe to alleviate symptoms of irritable bowel syndrome and recurrent chronic sinusitis or bronchitis (Cylactin^® ^and Fargo688^® ^(*E. faecium*) and Symbioflor 1 (*E. faecalis*)). To differentiate the two faces of this organism, genome-wide comparisons are necessary. Although hundreds of microbial genomes have been sequenced, only two *E. faecalis *genomes have been reported (V583 as a clinical isolate [4] and Symbioflor 1 as a commensal isolate [5]), but only the V583 genome has been made publicly available. In this strain, more than one-quarter of the genome is mobile DNA, more than any other sequenced bacterial genome [4]. The occurrence of multiple antibiotic resistance determinants in V583 [6] makes it difficult to manipulate genetically. Moreover, the vancomycin resistance phenotype makes this strain more of a risk to handle in the laboratory. To avoid these issues, most laboratories use strain OG1 or its close derivatives. OG1 is a human isolate subsequently shown to cause dental caries in rats [7]. OG1RF is a rifampicin and fusidic acid resistant derivative of OG1 [8,9]. By pulsed-field gel electrophoresis, Murray *et al*. [10] estimated the size of the OG1RF genome as 2,825 kb and created a restriction map of the chromosome. Multilocus sequence typing (MLST) showed that OG1RF is clonally distinct from V583 (differs in six out of seven alleles of housekeeping genes) [11] and characterization of regions flanking transposon insertions in OG1RF suggested that approximately 10% of their sequences differed [12].

OG1 and its derivatives have been successfully used over the past 20 years in various animal models, starting with the demonstration that it can cause caries in germ-free rats [7], and later to characterize factors important for *E. faecalis *virulence in a mouse model of peritonitis [13], a rabbit model of endophthalmitis [14], a rat model of endocarditis [15] and in a mouse urinary tract infection model [16]. OG1RF was also shown to be as virulent as V583 in the model host *Caenorhabditis elegans *[17]. In addition to its virulence, the main reasons for the extensive use of OG1RF as a laboratory strain are that it does not carry plasmids, is readily transformable by electroporation, and is not resistant to commonly used antibiotics, other than rifampicin and fusidic acid. These resistances were serially selected in OG1 to provide strain markers [9]. The lack of resistance to common antibiotics facilitates the selection of plasmids, transposons, and allelic replacement markers introduced into the strain.

Numerous factors important for virulence have been characterized in OG1RF. A recently described example are the Ebp pili, whose subunits are encoded by the *ebp *operon [18] and whose genes are regulated by EbpR [19]. A non-piliated mutant produces less biofilm than the parent strain and is attenuated in a rat model of endocarditis [18] and in a murine urinary tract infection model [16]. Also present is Ace, a member of the MSCRAMM (microbial surface component recognizing adhesive matrix molecules) family. The *ace *gene, like the *ebp *locus, is ubiquitous in *E. faecalis *and it occurs in at least four different forms that vary in the number of repeats of the B domain [20]. Ace mediates conditional (that is, after growth at 46°C or in the presence of serum or collagen) adherence of *E. faecalis *to collagen type IV and to laminin [21] and, in unpublished data, influences the ability of OG1RF to cause experimental endocarditis (KV Singh and BE Murray, unpublished observation). Finally, the Fsr system, a major positive and negative transcriptional regulator in OG1RF [22], affects expression of several virulence factor genes, including *gelE*, which encodes gelatinase [23], and contributes to infection in various animal models [15,24].

The distinct MLST profile and the wide range of phenotypic and genotypic analyses of OG1RF, including many molecular genetic studies and experiments in various animal models, suggested that genomic analysis of this strain would prove insightful and would be useful to future studies. Thus, we analyzed the sequence of *E. faecalis *OG1RF. This revealed approximately 232 kb encoding 227 open reading frames (ORFs) that are unique to this important strain compared to V583. The unique regions were then characterized further.

## Results and discussion

### General genome features

**Figure 1:**
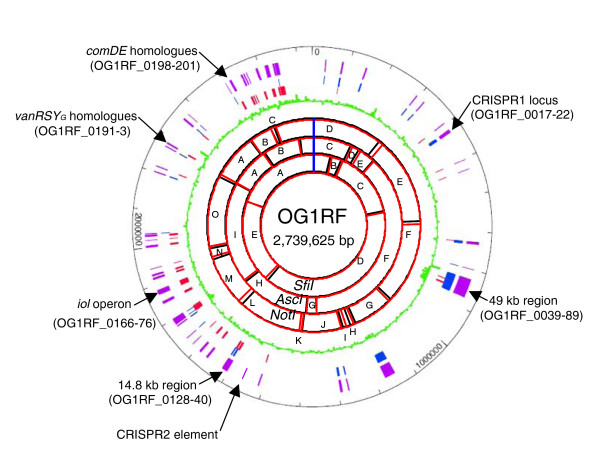
Map of the OG1RF chromosome. The following features are displayed (from the inside out): restriction maps using *Sfi*I, *Asc*I, and *Not*I (black) from Murray *et al*. [10] overlaid with the digestion profile predicted from the sequence (red); G+C content in percentage in green; the total OG1RF-unique genes are shown in purple with those in (+) orientation labeled in blue, and those in (-) orientation labeled in red.

The complete circular chromosome of OG1RF was found to be 2,739,625 bp with an average G+C content of 37.8%. The complete OG1RF sequence was obtained using three independent techniques (Solexa, the 454, and Sanger sequencing technique) with a higher than classic coverage (more than 100 times), diminishing the likelihood of sequencing-related frameshifts, base errors and/or misassembly. A comparison of our assembly of the closed OG1RF genome with the restriction map of OG1RF published by Murray *et al*. [10] showed only minor variations (primarily an overestimation of 30 kb for the *Sfi *I fragment E, 540 kb versus 509 kb predicted from the sequence; Figure 1).

**Figure 2:**
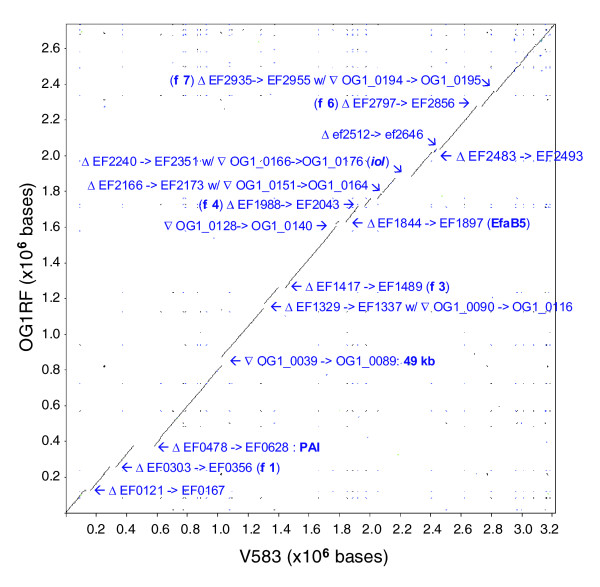
Dot plot of OG1RF versus V583 generated by BLASTN. The dot plot was generated by aligning the OR1RF genome against the V583 genome using BLASTN (e-value 1e-10). The alignment pairs were plotted according to their genome coordinates. The visible areas of divergences are labeled using 'Δ ' to indicate a sequence absent in OG1RF and '∇ ' to indicate a sequence unique to OG1RF (locus tag OG1_xxxx) when compared with V583 (locus tag EFxxxx). Phages 1, 3, 4, 5, 6, 7 of V583 (φ1 to 7; see [31]) and the PAI locations, all of which are missing from OG1RF, are also indicated.

We found 232 kb of OG1RF unique sequences distributed in 48 regions ranging from 101 bp to approximately 49 kb in length (Figure 1; Additional data file 1). Using the published DNA sequence of V583 as reference (NC_004668), OG1RF shares 2,474 ORFs as well as the 12 rRNA genes and 58 of 68 tRNA genes (Table 1). The 10 missing tRNA are localized in a region in V583 that has been replaced in OG1RF by a 49 kb region (see below). Surprisingly, the genomes align syntenically, as shown in Figure 2, despite the fact that 25% of the V583 genome is composed of mobile elements. Similarly, the presence of OG1RF-unique sequences has not affected the overall chromosomal arrangement. Some of the major insertions/deletions in the two genomes are shown in Figure 2, such as the absence of the pathogenicity island (PAI) in OG1RF and the presence of an approximately 49 kb fragment unique to OG1RF. However, most of the differences are small and cannot be visualized in this figure. Overall, we found 64 areas of divergence between the genomes that can be divided into 3 classes: an additional sequence present in OG1RF when compared with V583; a sequence replacement where a sequence in OG1RF differs from the sequence in V583; and the absence of a sequence from OG1RF when compared with V583.

**Table 1:** General features of OG1RF compared to V583

	V583		OG1RF
General features			
Size (base pairs)	3,218,031		2,739,633
G+C content (%)	37.5		37.8
			
rRNA genes	12		12
tRNA genes	68		58
			
Genes common to both strains		2,474*	
			
Genes unique to OG1RF			
Similar to known proteins			114^†^
Conserved hypotheticals			50
No database match			63
Total			227
			
Total number of ORFs	3,113		2,701^‡^

*The assessment of the genes common to both strains is based on the homology at the DNA level with the ORFs described for V583 (source TIGR [70]). The BLASTN cutoff e-value was 1e-5. ^†^This number includes the proteins with domain polymorphism (see text for details). ^‡^Estimated number of ORFs calculated by adding the OG1RF-unique ORFs to the number of ORFs shared with V583.

### CRISPR loci

**Figure 3:**
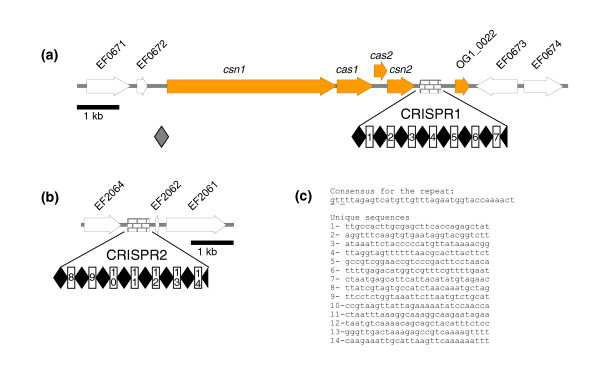
The two CRISPR loci of OG1RF. **(a)** The CRISPR1 locus. The CRISPR1 element is represented with a hatched box while the CRISPR1 associated genes are represented in orange; the white arrows indicate ORFs present in both OG1RF and V583. The black diamonds represent the 37 bp repeat sequences, while the open boxes with a number indicate the 29 bp unique sequences. **(b)** The CRISPR2 locus containing only a CRISPR element. **(c)** CRISPR consensus and unique sequences. The underlined bases indicate mismatches at these locations. The sequences numbered 1 to 14 represent the unique sequences located in the CRISPR1 and CRISPR2 elements.

The CRISPR (comprised of regularly interspaced short palindromic repeats) loci encoded by some bacterial strains is a recently described system that protects cells from infection with bacteriophage [25-27]. The specificity of the phage resistance conferred by the CRISPR elements and CRISPR-associated genes (*cas *genes) is determined by spacer-phage sequence similarity. OG1RF carries two CRISPR elements: CRISPR1 (between the OG1RF homologue of EF0672 and EF0673) and CRISPR2 (between the OG1RF homologue of EF2062 and EF2063); CRISPR1 is linked to *cas*-like genes while CRISPR2 is not (Figure 3). Both OG1RF CRISPR elements are composed of 7 repeats of a 37 bp palindromic sequence with a 29 bp spacer. None of the 29 bp spacers (14 total) have homology to any sequences in GenBank. The CRISPR1-associated proteins belong to the Nmeni subtype [28]. Species bearing this CRISPR/*cas *subtype have so far been found exclusively in bacteria that are vertebrate pathogens or commensals. The Nmeni subtype is characterized by the presence of four specific *cas *genes and a single copy of the repeat that is upstream of the first gene in the locus. The four *cas *genes encode Cas_csn1 (possible endonuclease), Cas1 (novel nuclease), Cas2 (conserved hypothetical protein), and Cas_csn2 (conserved hypothetical protein). The repeat upstream of *cas_csn1 *appears to have degenerated since it shares only 23 bp with the 37 bp repeat cluster downstream of the last gene. A unique feature of the OG1RF CRISPR1 locus is the presence of a gene downstream of the element, which encodes a hypothetical 119 amino acid transmembrane protein.

The presence of the CRISPR loci among *E. faecalis *strains may be a powerful tool to avoid the load of prophage replication. To determine the distribution of the CRISPR1 locus in *E. faecalis *strains, 16 isolates of various MLST types were tested for the presence (PCR with primers specific for *csn1 *and *cas1*) or absence (PCR with primers overlapping the junction between EF0672 and EF0673) of the CRISPR1 locus (Table 2). Seven strains were *cas *positive, but negative for the junction and the remaining nine were positive only for the junction. This indicates that the location of the CRISPR1 locus appears to be conserved (between EF0672 and EF0673 when compared with the V583 genome). Interestingly, the two vancomycin resistant strains tested were both *cas *negative. It is appealing to postulate that the presence of the CRISPR locus in OG1RF may be the reason for the absence of prophage in this strain.

**Table 2:** Frequency of the CRISPR locus among *E. faecalis*

Name	Other	Origin	Source/reference	MLST	ErmR*	VanR^†^	cas^‡^	EF0672-3^§^
TX4002	OG1RF	Human	[8,9]	1	-	-	+	-
TX2708	V583	Clinical isolate	[6]	6^¶^	+	+	-	+
TX2144	E1840	Clinical isolate	Ruiz-Garbajosa P.^#^	40	+	-	+	-
TX2135	E1795	Hospital survey	Ruiz-Garbajosa P.	44	-	-	-	+
TX2137	E1798	Hospital survey	Ruiz-Garbajosa P.	16	+	-	+	-
TX2141	E1825	Clinical isolate	Ruiz-Garbajosa P.	25	-	-	-	+
TX2140	E1803	Hospital survey	Ruiz-Garbajosa P.	38	-	-	-	+
TX2138	E1801	Hospital survey	Ruiz-Garbajosa P.	48	-	-	-	+
TX2146	E1844	Clinical isolate	Ruiz-Garbajosa P.	61	-	-	-	+
TX2139	E1802	Hospital survey	Ruiz-Garbajosa P.	35	+	-	+	-
TX4240	A0826	Pig	Jensen L.	98	+	-	+	-
TX4247	E1876	Pig	Gaastra W.	20	+	-	+	-
TX4245	E1872	Dog	Gaastra W.	16	+	-	+	-
TX4243	E0252	Calf	Mevius D.	23	+	+	-	+
TX4255	A0808	Clinical isolate	Kawalec M.	88	-	-	-	+
TX4259	A1006	Clinical isolate	Kawalec M.	135	-	-	-	+

*Erythromycin resistance was tested at 5 μg/ml. ^†^Vancomycin resistance was tested at 10 μg/ml. ^‡^Two sets of primers were used to detect the *cas *genes (*cas1 *and *csn1*). ^§^This set of primers amplifies the junction between EF0672 and EF0673 where the CRISPR1 locus is inserted in OG1RF. ^¶^CC2. ^# ^Ruiz-Garbajosa P. (Spain), Jensen L. (Denmark), Gaastra W. and Mevius D. (Netherland), and Kawalec M. (Poland).

### A 14.8 kb region inserted in the 23.9 kb region containing *fsrA *and *fsrB*

Nakayama *et al*. [29] described a conserved 23.9 kb chromosomal deletion when comparing *fsrA-*lacking/*fsrC*^+^/*gelE*^+ ^strains (by PCR) from various origins with V583; the deleted sequences start in the middle of EF1841, include the *fsrAB *genes and end in the middle of the *fsrC *gene (EF1820). Loss of the *fsr *regulatory components results in a gelatinase-negative phenotype under routine test conditions despite the fact that these strains still carry the *gelE *gene [23,29]. The absence/presence of the 23.9 kb region, from EF1820/*fsrC *to EF1841, did not appear to correlate with the clinical origin of the isolates [30]. In a more recent analysis of relationships between various *E. faecalis *strains, the 23.9 kb region was not detected in 86% of the strains of the clonal complex (CC)2, 58% of the CC9 strains, nor in any of the CC8 strains [31]. The Symbioflor 1 strain, used as a probiotic, is one representative of the 7.4% of *E. faecalis *isolates that are missing the *gelE *gene in addition to the 23.9 kb region [5,30]. Our analysis of this area in OG1RF revealed the presence of an additional 14.8 kb fragment inserted between the corresponding EF1826 and EF1827 of OG1RF (confirmed by PCR; results not shown). In OG1RF, this 14.8 kb region contains two loci, a WxL locus (described below) and a seven-gene locus that may encode a possible ABC transporter with similarity to one annotated in *Pediococcus pentosaceus*.

### Components of the cell surface

It has been shown in *E. faecalis *that at least one cell surface protein (Ace) is subject to domain variation [20] and it has been postulated that domain variation may help bacteria escape the immune system. We found more polymorphisms in two families of *E. faecalis *proteins present on the cell surface: the MSCRAMMs and the WxL domain surface proteins. The MSCRAMMs are composed of two large regions, namely, the non-repeat A region (which is usually the ligand binding region for extracellular matrix molecules such as collagen or fibrinogen) and the B region (which typically contains repeated sub-domains). The B region of Ace contains five repeats in OG1RF, while it contains only four in V583 [20]. We found two other MSCRAMM proteins that show polymorphisms in the number of their B repeats. OG1RF_0186 (corresponding to EF2505 of V583) has four repeats compared to seven in V583, and OG1RF_0165 (corresponding to EF2224 of V583) has eight repeats compared to five in V583. It has been proposed that the repeats are used as a stalk that projects the A region across the peptidoglycan and away from the cell surface [32]. A hypothesis that the number of repeats may be proportional to the depth of the peptidoglycan has been proposed [32]. However, OG1RF_0186 carries fewer repeats than EF2505 while Ace and OG1RF_0165 carry more repeats than their counterparts in V583, suggesting that our observation does not fit this hypothesis or that the peptidoglycan depth is not uniform. Apart from these three MSCRAMMs with B-repeat polymorphisms, we identified two unique MSCRAMM proteins in OG1RF: a homologue of EF0089 (OG1RF_0063, which shares 48% similarity) and a homologue of EF1896 (OG1RF_0039, which shares 75% similarity); both are located in the approximately 49 kb region unique to OG1RF described below (Figure 1; Additional data file 1).

Another family of *E. faecalis *surface proteins includes the newly described WxL domain surface proteins. Siezen *et al*. [33] reported a novel gene cluster encoding exclusively cell-surface proteins that is conserved in a subgroup of Gram-positive bacteria. Each gene cluster has at least one member of three gene families: a gene encoding a small LPxTG protein (approximately 120 amino acids); a gene encoding a member of the DUF916 transmembrane protein family; and a gene encoding a WxL domain surface protein. In addition, members of these gene families were found as singletons or associated with genes encoding other proteins (Additional data file 2). Recently, it was shown that the WxL domain attaches to the peptidoglycan on the cell surface [34] and one member of this WxL domain family, the homologue of EF2686 in OG1RF (a probable internalin protein), was shown to be important for virulence in a mouse peritonitis model and is required for dissemination to the spleen and liver [35]. OG1RF shares five complete WxL loci with V583 (EF0750-7, EF2682-6, EF2970-68, EF3181-8, and EF3248-53). OG1RF does not contain homologues of EF2248-54 (carrying instead the *iol *operon), though it has a novel WxL locus within the 14.8 kb unique region upstream of the *fsr *locus (Additional data file 2). In addition to the variation in the number of WxL loci, we also observed polymorphisms in six of the WxL domain surface proteins. For example, OG1RF_0213 shares 88% similarity with EF3188, while OG1RF_0224, OG1RF_0225, and OG1RF_0227 share 64-68% similarity with their V583 counterparts, EF3248, EF3250, and EF3252, respectively. Also, in place of EF3153, EF3154, and EF3155 (which share 70% similarity among themselves), were found non-distantly related homologues, OG1RF_0209 and OG1RF_0210, which share 60-80% similarity with EF3153, EF3154, and EF3155. It is interesting to note that while several of these WxL loci, including the EF0750 and EF3184 loci, were present by hybridization in all the strains (clinical or food isolates) tested by Lepage *et al*. [36], other loci, including the EF3153 and EF3248 loci, were not detected in the majority of these strains. In addition, it appears that the EF3248 locus diverges in the Symbioflor 1 strain. When compared to V583, the sequence identity in this area between the two strains appears to be as low as 75% (depicted in Figure 2 from reference [5]).

However, because the Symbioflor 1 genome sequence is not currently available, it was not possible to compare their respective sequences in more detail. Since these proteins are located at the surface of the cell, the low level of homology shared between them may be the result of antigenic variation. More analyses are required for a better understanding of the number, frequency and function of these WxL domain proteins and their possible relationship with the diversity of *E. faecalis*.

Finally, as previously found using PCR, the *cpsCDEFGHIJK *operon capsule polysaccharide genes [37] were confirmed here as missing, although OG1RF carries the *cpsA *and *cpsB *genes, which were proposed to be essential for *E. faecalis *since all strains tested by Hufnagel *et al*. [37] carry these two genes. In OG1RF, the region that would encode the *cps *operon is only 59 bp in length and has no homology with V583. Thus, while V583 and OG1RF share much similarity between their surface components, there are unique differences that could potentially be important in affecting the behavior of the strains and might be useful for strain typing.

### Two-component regulatory systems

**Figure 4:**
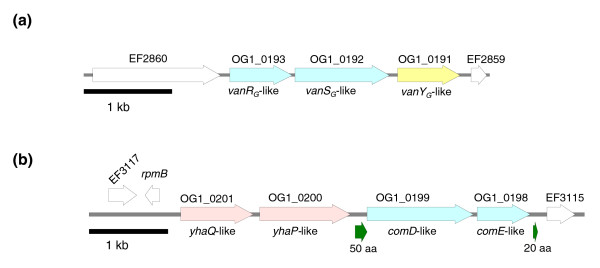
Two-component systems unique to OG1RF. **(a)** Two-component system with homology to the Van_G _system. **(b) **Two-component system with homology to the *comCD *genes of *S. pneumoniae*. The two-component system (OG1RF_0198 and OG1RF_0199) is indicated in light blue; the two ORFs encoding potential transporter proteins (OG1RF_0200 and OG1RF_0201) are represented in pink. In green are indicated two small ORFs encoding polypeptides of less than 51 amino acids. The white arrows indicate ORFs also present in V583.

OG1RF lacks four two-component systems found in V583. These are histidine kinase-response regulator (HK-RR)08, HK-RR12 located in the PAI, HK-RR16 and the *vanB *regulatory system HK-RR11 [38]. However, an OG1RF-unique two-component system with high homology with the *van*_G _locus was found at the location corresponding to the region between EF2860 and EF2861 in V583 (Table 3). OG1RF_0193 shares 82% similarity with VanR_G _and 81% similarity with VanR_G2_. Similarly, OG1RF_0192 shares 68% similarity with VanS_G _and VanS_G2_. A gene (OG1RF_0191) encoding an M15 family muramoyl pentapeptide carboxypeptidase is located downstream of these two-component regulatory genes (Figure 4a). The predicted carboxypeptidase (OG1RF_0191) shares 69% similarity over 179 amino acids with EF2297, a membrane-associated D, D-carboxypeptidase encoded by the *vanB *operon in V583. However, OG1RF_0191 lacks an identifiable transmembrane domain that is important to the VanY function and it is likely, therefore, that this protein may be a soluble D, D-carboxypeptidase/transpeptidase as seen in *Streptomyces *[39] and *Actinomadura *[40], and thus may not be involved in peptidoglycan metabolism. Consequently, it seems unlikely that this operon is a remnant of a vancomycin resistance operon in OG1RF, but rather part of a still unknown regulatory pathway.

**Table 3:** OG1RF-unique regulators

OG1RF	Description	Best hit	Size*	Comments
OG1RF_0070	Transcriptional regulator	116512576	102	-
OG1RF_0073	LytR family response regulator	81428169	151	-
OG1RF_0120	BglG family transcriptional antiterminator	47095712	494	Probable regulator of the downstream PTS system
OG1RF_0138	Transcriptional regulator	116493423	219	Probable transcriptional regulator of the downstream ABC superfamily transporter
OG1RF_0143	GntR family transcriptional regulator	82745913	236	Probable regulator of the downstream PTS system
OG1RF_0175	DNA binding protein	15890504	293	Probable regulator of the *iol *operon
OG1RF_0192	Sensor histidine kinase VanS_G_	119635646	371	Best homology with Van_G _and
OG1RF_0193	Response regulator VanR_G_	119635645	235	Van_G2 _two-component systems.
				OG1RF_0192 and OG1RF_0193 appear cotranscribed with a gene encoding a M15 family muramoylpentapeptide carboxypeptidase
OG1RF_0198	Response regulator	47567135	240	Best homology with AgrA from *Bacillus cereus *G9241. However, no presence of AgrB or AgrD homologues in the vicinity. Also similar to ComE of *S. pneumoniae *(52% similarity)
OG1RF_0199	Sensor histidine kinase	47567134	443	Best homology with AgrC from *Bacillus cereus *G9241. Also similar to ComD of *Streptococcus pneumoniae *(48% similarity)
OG1RF_0220	Probable endoribonuclease MazF	69244828	114	Toxin-antitoxin described in *E.*
OG1EF_0221	Probable antitoxin MazE	69244829	77	*coli *and recently on an *E. faecium *plasmid

*Amino acids

### The *iol *operon

**Figure 5:**
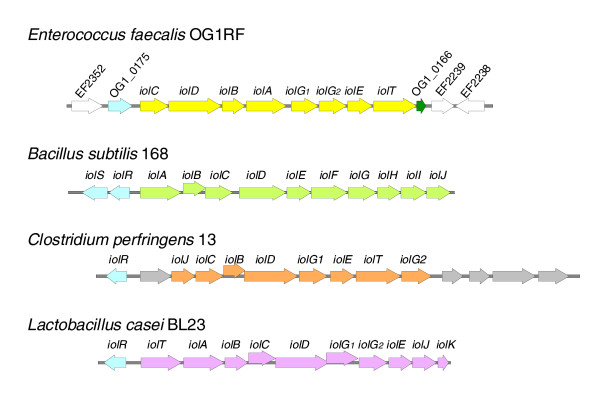
The *iol *operon. The *iol *genes are labeled based on the homology/conserved motif of their encoded proteins with known enzymes necessary for myo-inositol degradation. For all strains, the described or probable regulator is represented in blue. *E. faecalis *OG1RF: the *iol *operon is represented in yellow, OG1RF_0166 (green arrow) located downstream of the *iol *operon encodes a probable PTS IIC component, while the white arrows indicate ORFs also present in V583. For *B. subtilis *168, *C. perfringens *strain 13, and *L. casei *BL23, the *iol *genes are represented in green, orange and purple, respectively. *C. perfringens iol *mRNA transcript includes five other genes encoding proteins whose functions do not appear to be related to myo-inositol degradation; these genes are represented in gray.

OG1RF carries an *iol *operon while V583 does not. This operon encodes the factors necessary for the degradation of myo-inositol into glyceraldehyde-3P. Many soil and plant micro-organisms, including *Bacillus subtilis *[41] (first *iol *operon identified), *Klebsiella *spp. [42], and cryptococci [43], have been reported to use myo-inositol as a sole carbon source. Myo-inositol, one of the nine isomers of the inositol group, belongs to the cyclitol group and is abundant in nature, particularly in the soil. The OG1RF *iol *operon appears to be closely related to ones described in *Clostridium perfringens *[44] and *Lactobacillus casei *[45]. In *L. casei*, the myo-inositol operon consists of ten genes with an upstream divergent regulator gene, *iolR*. In OG1RF, the operon appears to include ten genes, beginning with a probable transcriptional regulator (helix-turn-helix domain protein). Also, the OG1RF operon carries two copies of an *iolG*-like gene, which encodes inositol 2-dehydrogenase, the first enzyme of the myo-inositol degradation pathway (Figure 5). However, the order of the genes is not the same between *E. faecalis *and *L. casei*. In addition, *iolH*,*iolJ *and *iolK*, present in *L. casei*, are not present in OG1RF, nor are *iolH *and *iolK *present in the *C. perfringens iol *operon.

Yebra *et al*. reported that *L. casei *was the sole member of the Lactobacillales to carry a functional *iol *operon [45]. To survey *E. faecalis*, also a member of this order, for the presence of the *iol *operon, 48 isolates with different MLST and/or from various origins (including OG1RF and V583) were tested for myo-inositol fermentation; 23 of 48 isolates were positive. In addition, PCR verified the presence of *iolE *and *iolR *in these strains and in one negative for myo-inositol fermentation, indicating that the *iol *operon is not unique to OG1RF. To verify that the *iol *genes are responsible for the fermentation of myo-inositol in OG1RF, transposon insertion mutants [9] in the *iolB *and *iolG2 *genes of OG1RF were tested. Both mutants failed to ferment myo-inositol (data not shown), demonstrating that these genes are essential for myo-inositol fermentation. To investigate whether the *iol *operon was 'inserted into' or 'removed from' a putative ancestral strain, the sequences surrounding the *iol *genes were examined. In OG1RF, the *iol *operon is located between the equivalent of EF2239 and EF2352 when compared with V583. In V583, this region encodes probable prophage proteins and carries the *vanB *transposon, which confers vancomycin resistance. Since we did not identify any remnants of the *iol *operon in V583, it would appear that at least two independent events at the same location differentiate OG1RF and V583, suggesting that it is a hot region for rearrangement. This region between EF2239 and EF2352 (111 Kb) is also missing in the Symbioflor 1 strain (referred to as gap 2) [5]. The possible junction and presence of unique sequence in this region, if investigated, was not mentioned in the publication. Nonetheless, preliminary analysis of other strains' genotypes in this area seemed to confirm the hypothesis of a hot region for rearrangement (data not shown).

### A homologue of Tn*916 *in OG1RF

An analysis of the G+C content of OG1RF unique regions revealed several loci with a lower G+C content than the 37.8% average content of OG1RF. One of these is an approximately 49 kb fragment with a G+C content of 32.1% located between an rRNA operon and the homologue in OG1RF of EF1053, replacing 10 tRNA genes present in V583 (Figure 1). This fragment appears to be a patchwork composed of hypothetical genes, homologues of Tn*916*-associated genes and homologues of genes from other Gram-positive organisms, including *Listeria*, *E. faecium*, staphylococci, or lactococci (Additional data file 1). It is interesting to note that this region contains: a putative adhesin protein gene (OG1RF_0039) at one end of the fragment; homologues of 14 Tn*916*-associated genes (Tn*916*_2 to Tn*916*_12, Tn*916*_18 and Tn*916*_19, with an average of 70% similarity); and a gene encoding a putative integrase (OG1RF_0088) at the other end - these three features are also present in Tn*5386 *in *E. faecium *D344R [46]. However, the approximately 49 kb fragment lacks an excisase gene and the probable lantibiotic ABC transporter genes present in Tn*5386*.

### An uninterrupted competence operon in OG1RF

**Figure 6:**
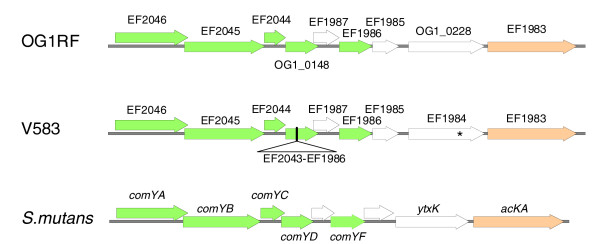
The OG1RF competence operon and its similarity with the competence operon of *S. mutans*. The ORFs essential for natural competence in *S. mutans *are shown in green as well as their homologues in OG1RF and V583. The ORF corresponding to the homologue of ComYD was not described in V583 [4], due to the presence of a probable prophage (EF1986-EF2043). The premature stop codon in EF1984 in V583 is indicated with an asterisk. *ackA/*EF1983 is represented in orange. The proteins encoded by the ORFs represented in white do not share any features of the known competence proteins or homology between *S. mutans *and *E. faecalis*; in *S. mutans*, *ackA *and *ytxK *are co-transcribed with the *comY *genes [47].

OG1RF contains what appears to be an intact competence operon while that of V583 appears to be non-functional. This operon in OG1RF is similar to a nine-gene operon described in *Streptococcus mutans *[47], as shown in Figure 6. For example, the homologue in OG1RF of EF2046 shares 61% similarity with ComYA and the OG1RF homologue of EF2045 is 55% similar to ComYB. In *S. mutans*, only the first seven genes of the operon are essential for competence [47]. In V583, the fourth gene of this operon (corresponding to OG1RF_0148) is interrupted by phage 4 (EF1896-EF2043); in addition, EF1984 contains a premature stop codon not found in the corresponding gene in OG1RF (OG1RF_0228).

Natural competence has not been reported for *E. faecalis*. To assess the functionality of this operon in OG1RF, we evaluated the competence of cells in different phases of growth (early log growth to stationary phase) using pAM401 [48] and pMSP3535VA [49]. We were not able to show natural competence under the conditions tested. We have also noted that V583 is less transformable by electroporation than OG1RF. To investigate the possibility that directly or indirectly the *com *operon might be responsible for this phenotype, we also evaluated transformability by electroporation. When compared with OG1RF, transposon mutants [12] in the OG1RF equivalent of EF2045 (encoding the *comGB *homologue) and in the OG1RF equivalent of EF1986 (encoding the *comGF *homologue) showed similar levels of transformability by electroporation (data not shown), implying that the difference in electroporation efficiency observed between OG1RF and V583 is not related to this locus.

In *Streptococcus pneumoniae *[50], the competence operon is tightly regulated by a quorum sensing two-component system (ComDE) and a competence-stimulating peptide (CSP; encoded by *comC*). We did not find any homologues of CSP in OG1RF. Two homologous ComDE sensor histidine kinase/response regulators were found in OG1RF, one of which is FsrC/FsrA. Based on our previous microarray data, the Fsr system does not regulate the *comY *operon, at least under our previously used conditions (mid-log phase growth to early stationary phase in brain heart infusion (BHI)) [22]. The other ComDE homology is that with a two-component system unique to OG1RF (OG1RF_0199 and OG1RF_0198, respectively) that lies on a 4,706 bp unique fragment that maps between EF3114 and EF3115 in V583. This fragment also carries two genes (OG1RF_0200 and OG1RF_0201) encoding homologues of the YhaQ and YhaP sodium efflux ATP-binding cassette efflux/transporter proteins (Figure 4b). Although they are potential elements of a secretion apparatus, these two proteins do not share any homology at the protein level with the competence secretion apparatus ComAB of *S. pneumoniae *[51] nor CslAB from *S. mutans *[52]. Searching for a possible CSP in the vicinity of these genes, we identified a small ORF encoding 50 amino acids between *yhaP *and OG1RF199 and another encoding 20 amino acids downstream of OG1RF198. More analysis will be required to determine if there are conditions in which the OG1RF *com *operon is expressed and to determine whether or not this two-component system is involved in competence.

### Limited presence of mobile elements

By probing a microarray of the V583 genome and plasmids with OG1RF genomic DNA, we previously estimated that only 75% of V583 ORFs were also present in OG1RF [22]. Later, Aakra *et al*. [53] compared nine strains, including OG1RF to V583, using comparative genomic hybridization. In these results, OG1RF appears to carry a few genes included in the PAI, and a few prophage genes. Using the complete genome sequence, we have now found that OG1RF lacks 639 genes and the three pTET plasmids described in V583. All but 45 of the missing genes are associated with putative mobile elements, such as the entire PAI, the recently described phages 1, 3, 4, 5, 6, and 7 [31], and the approximately 111 kb area between genes EF2240 and EF2351 (including the *vanB *transposon) present in V583. The absence of these elements appears also to be a characteristic of the commensal strain Symbioflor 1, although because the genome was not completely finished, the possibility remains that some of these regions were not sequenced. In conclusion, other than the approximately 49 kb fragment containing a Tn*916 *homologue, it appears that OG1RF has only one additional possible mobile element derivative, namely the phage 2 proposed to be part of the core genome [31].

### Fusidic acid and rifampicin resistance

OG1RF was sequentially selected from OG1 for resistance to fusidic acid and rifampicin [9]. The mutation leading to rifampicin resistance was identified in the *rpoB *gene by Ozawa *et al*. [54] and is caused by the A1467G mutation, which results in substitution of arginine for histidine at amino acid 489. The mutation also affected the clumping phenotype of *traA *mutants and this effect appears to be specific for the pAM373 system [54]. All of the other 22 differences in *rpoB *between OG1RF and V583 are synonymous. Fusidic acid resistance is associated with mutation(s) in the *fusA *gene, which encodes elongation factor G. We compared *fusA *from OG1RF with that in V583 and identified two differences (C1368A and T1992C). The mutation T1992C is synonymous, while the mutation C1368A leads to the presence of glutamine (histidine in V583) at position 404 in OG1RF. Mutations in this region have been associated with fusidic acid resistance in *Staphylococcus aureus *[55,56], and thus the C1368A mutation is likely the cause of the fusidic acid resistance phenotype in OG1RF.

### Virulence and biofilm comparisons of OG1RF with V583

**Figure 7:**
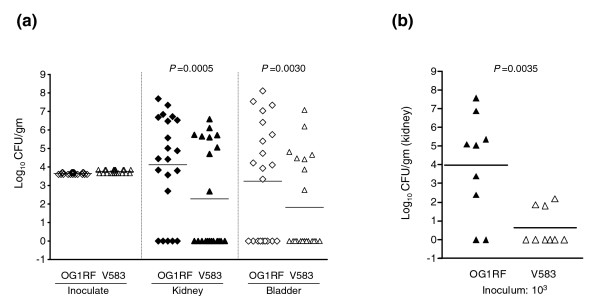
Comparison of OG1RF and V583 in a mouse urinary tract infection model. **(a)** Mixed infection by wild-type *E. faecalis *strains OG1RF and V583 in the kidneys and urinary bladders of mice (n = 21; competition assay). Data are expressed as the log_10_(CFU)/gm for OG1RF or V583; the log_10_(CFU)/gm for both kidneys were combined and averaged from two independent experiments. Black solid diamonds and triangles represent *E. faecalis *strains OG1RF and V583, respectively, for kidney homogenates, and empty diamonds and triangles represent OG1RF and V583, respectively, for urinary bladder homogenates. Horizontal bars represent geometric means. Log_10_(CFU) were compared for statistical significance by the paired *t*-test. The minimum detection limit in these experiments was 10^1 ^and 10^2 ^CFU/gm of kidney and urinary bladder homogenates, respectively. **(b) **Mono-infection using *E. faecalis *strains OG1RF or V583 in the kidneys of mice (10^3 ^CFU per mice, n = 9). Data are expressed as log_10_(CFU)/gm for OG1RF recovered from kidney homogenates 48 h after infection; the log_10_(CFU)/gm for a kidney pair were combined and averaged. Black and white triangles represent OG1RF and V583, respectively. Horizontal bars represent geometric means. The CFU of V583 recovered from kidneys was significantly reduced compared to the CFU of OG1RF, as determined by the unpaired *t*-test.

When compared in the mouse peritonitis model, the LD_50 _values of V583 in different determinations were lower (4.8 × 10^7 ^to 1.1 × 10^8 ^colony forming units (CFU)/ml) than the LD_50 _values of OG1RF (1.2 × 10^8 ^to 4.8 × 10^8 ^CFU/ml). However, at comparable inoculum, OG1RF (4 × 10^8 ^CFU/ml) showed more rapid mortality versus V583 (5 × 10^8 ^CFU/ml) in the first 48 hours (*P *= 0.0034; Additional data file 3). In a urinary tract infection model administering mixed equal inocula of V583 and OG1RF, OG1RF significantly outnumbered V583 in kidney with geometric means of 1.3 × 10^4 ^CFU/gm for OG1RF versus 1.9 × 10^2 ^CFU/gm for V583 (*P *= 0.0005); in urinary bladder homogenates, the geometric mean CFU/gm was 1.7 × 10^3 ^for OG1RF versus 6.6 × 10^1 ^for V583 (*P *= 0.003; Figure 7a). Similarly, in mono-infection, the geometric mean CFU/gm of OG1RF in kidneys was 9.4 × 10^3 ^versus 4 × 10^1 ^for V583 (*P *= 0.0035; Figure 7b). We also found that OG1RF produced 20% more biofilm (*P *< 0.01) than V583 at 24 hours (results not shown). These results, together with the previous results in *C. elegans *[17], demonstrate that OG1RF, although lacking what was thought to be important for virulence (PAI, plasmids, prophages), is as pathogenic as V583 in at least three assays.

## Conclusion

*E. faecalis *OG1RF carries a number of unique loci compared to V583. Those of particular interest include new surface proteins (MSCRAMMs and WxL domain proteins), an operon encoding myo-inositol utilization, an intact competence operon, and two CRISPR elements. The CRISPR elements may be of particular significance when one considers that most of what is missing from OG1RF compared to V583 consists of mobile genetic elements (MGEs), including 6 phages or remnants thereof. The presence of the CRISPR elements in OG1RF provides a tantalizing, but as yet unproven, explanation for the discordance in the number of mobile elements between these two strains.

The acquisition of MGE is believed to be an important mechanism by which the species *E. faecalis *had been able to generate genetic diversity and, thereby, highly variable phenotypes [4]. It has been proposed that the ability of *E. faecalis *to cause healthcare related infections is associated with these MGEs [4,5]. This hypothesis was supported by several studies that have highlighted the importance of virulence determinants carried by these mobile elements, such as cytolysin [57] by the PAI. However, more recent results from Aakra *et al*. [53] and Lepage *et al*. [36] demonstrate that these factors may be present in harmless strains while absent in clinical isolates, indicating that *E. faecalis *virulence is not dependent on any single virulence factor. Indeed, few studies have compared the virulence pattern of strains from various origins. The increased ability of OG1RF to infect kidneys and to produce biofilm, despite the absence of MGEs and their associated virulence factors, was surprising. Different possibilities can be proposed relating to the factors important for these differences in enterococcal infections. One of these is that virulence in the assays used may be linked to the shared core genome of these two strains, with the differences arising from the unique genes. On the other hand, virulence could be associated primarily with the genes unique to each strain, but with each set being able to complement the absence of the other. It seems most likely that virulence, and/or some combination of virulence and fitness, is caused by the expression of a mixture of both the core and unique genes. It is also important to remember that *E. faecalis *is a well adapted commensal, carrying the genes necessary to survive and to colonize the gut, and that a subset, particularly MLST CC2 and CC9 [58], predominates among hospital acquired infections. It may be that these clonal complexes are not more virulent *per se*, as defined in the assays described here, but rather are better able to survive and/or colonize hospitalized patients, taking advantage of factors that predispose to nosocomial infections such as urinary or venous catheters, or mucositis, among others.

Sequencing of more *E. faecalis *strains may facilitate our understanding of the path from commensalism to pathogenicity, a crucial prerequisite for designing therapeutic interventions directed to control an organism that is already resistant to a large spectrum of antibiotics.

## Materials and methods

### Strains

*E. faecalis *OG1 is a strain of human origin (formerly designated 2SaR [7]) and was subsequently selected on rifampicin and fusidic acid [8,9] to generate OG1RF (deposited at the American Type Culture Collection (ATCC) under ATCC accession number 47077). V583 is a vancomycin resistant *E. faecalis *strain [6], recovered from a blood culture of a patient hospitalized at the Barnes Hospital, St Louis, MO, USA in February 1987 (ATCC accession number 700802, NCBI complete genome accession number NC_004668). Bacteria were grown routinely at 37°C in BHI broth (Difco Laboratories, Detroit, MI, USA) or BHI agar unless otherwise indicated. Comparisons of OG1RF and V583 grown in broth (BHI, tryptic soy broth with glucose (TSBG), or BHI with 40% serum) did not reveal any obvious differences.

### DNA sequencing and genome assembly

Genomic DNA was purified from cesium chloride (CsCl) gradients of whole cell lysates [10]. DNA sequencing was performed by a combined approach using 454 Life Sciences pyrosequencing strategies [59] and the Solexa approach [60]. Read-pair information was used to create higher order scaffolds. Sanger sequencing was used for OG1RF whole gun sequencing and finishing. The coverage was 28× by the 454, 104× by Solexa, and 4.5× by Sanger sequencing technique. The assembly was done using Atlas [61].

### Gene identification and annotation

Gene prediction and manual annotation were performed as previously described [62]. Glimmer [63] and GeneMark [64] were used independently to predict ORFs. Visualization of gene predictions was performed using the Genboree system [65] and the CONAN database [66]. OG1RF-unique ORFs were analyzed with BLASTN and BLASTX. Protein sequences were analyzed by BLASTP versus the nr database at NCBI [67]. When appropriate, other predictive tools were used as described previously [62]. This whole genome shotgun project has been deposited at DDBJ/EMBL/GenBank under the project accession ABPI00000000. The version described in this paper is the first version, ABPI01000000. This project includes also the annotation of the ORFs unique to OG1RF. The OG1RF-unique ORFs are listed in Additional data file 1.

### Transposon mutations in OG1RF-unique sequences

Following the creation of an *E. faecalis *Tn*917 *library [12], 6,237 sequences representing the flanking regions of the transposon insertion sites were obtained and compared to the V583 genome by BLASTN. A total of 196 sequences were unique to OG1RF. Thirty-seven of the unique genes contained a transposon insertion. The locations of the transposon insertions are listed in Additional data file 1.

### Carbohydrate fermentation tests

Forty-eight *E. faecalis *isolates, including OG1RF and V583, having different MLST profiles, pulsed field gel electrophoresis types or from various geographical origins, were streaked onto BHI agar and incubated overnight at 37°C. Five to ten colonies of each strain were resuspended in 100 μl of 0.9% saline in a microtiter plate and tested for fermentation using BBL™ Phenol Red Broth Base (Diagnostic Systems, Sparks, MD, USA) supplemented with agar and either 10 mM glucose (positive control), 10 mM dulcitol (negative control), or 10 mM myo-inositol (Sigma, St Louis, MO, USA). Plates were read after incubation at 37°C for 24 h; a yellow halo around the colony was considered positive for fermentation. *iolB *and *iolG2 *transposon mutants [12] were also tested.

### PCR

PCR was performed using genomic DNA purified using Bactozol™ (Molecular Research Center, Inc., Cincinnati, OH, USA), as recommended by the manufacturer. Specific PCR primer pairs (Additional data file 4) were used to assess the presence of the OG1RF-unique sequences and for confirmation of flanking DNA regions in common with V583.

### Competence assays

To test strains for competence, overnight cultures, grown at 37°C in Todd-Hewitt broth, were diluted in Todd-Hewitt broth to an OD_600 nm _of 0.05 and then further diluted 10,000-fold in Todd-Hewitt broth to a final volume of 100 ml. After 30 minutes at 37°C, with shaking at 150 rpm, and every hour for 10 h, 0.5 ml samples were removed and 2.5 μg of plasmid DNA were added. The plasmids tested were pAM401 [48] and pMSP3535VA [49]. The samples were incubated for 2 h before plating on BHI or BHI plus antibiotic (chloramphenical 10 μg/ml for pAM401 or kanamycin 2 mg/ml for pMSP3535VA). Following overnight incubation at 37°C, the total numbers of CFU/ml recovered on selective agar for the plasmid were compared to the total number of CFU/ml (plated on BHI agar) for each time point.

### Biofilm assay and statistical analysis

The biofilm assay was performed as described by Mohamed *et al*. [68]. Each assay was performed using 16 wells on three occasions. The median was calculated using the 48 OD_570 nm _readings on data pooled from all experiments and statistical analysis was performed using a non-parametric *t*-test.

### Mouse peritonitis model

*E. faecalis *strains OG1RF and V583 [6] were tested using a previously published method [13]. Mice were injected intraperitoneally with appropriate dilutions of premixed bacteria/sterile rat fecal extract and were observed for five days. Two-fold dilutions of test bacteria in the range 10^7^-10^9 ^CFU were used as the inocula for LD_50 _determination using 6-9 mice per inoculum group. Inocula CFU geometric mean values were obtained and used for LD_50 _calculation by the method of Reed and Muench [69].

### UTI model for competition assay and ID50 determination

*E. faecalis *strains OG1RF and V583 were tested in the UTI model as previously described [16]. For the mixed inoculum experiments, an approximately 1:1 ratio of *E. faecalis *OG1RF:V583 at approximately 10^3 ^CFU each was used. Two independent experiments, using 10 and 11 mice, respectively, were performed and the results were combined. The log_10_(CFU) of OG1RF and V583 per gram of tissue of each animal (kidney or bladder) from mixed infection were analyzed for significance by the paired *t*-test. For mono-infection, approximately 10^3 ^CFU organisms grown in BHI + 40% horse serum were used for each strain independently and CFU obtained from kidney pairs (nine mice per strain) were analyzed for significance by the unpaired *t*-test. The minimum detectable limits of recovered bacteria were 10^1 ^and 10^2 ^CFU/gm of kidney pairs and urinary bladder homogenates, respectively.

## Abbreviations

ATCC, American type culture collection; BHI, brain heart infusion; CC, clonal complex; CFU, colony forming units; CRISPR, comprised of regularly interspaced short palindromic repeats; CSP, competence-stimulating peptide; HK-RR, histidine kinase-response regulator; MGE, mobile genetic element; MSCRAMM, microbial surface component recognizing adhesive matrix molecules; MLST, multilocus sequence typing; ORF, open reading frame; PAI, pathogenicity island.

## Authors' contributions

GMW, DAG, and BEM designed the study. AB performed much of the post-annotation analysis and non-animal experiments, and wrote the draft of the manuscript. KVS performed the animal experiments. AB, DAG, XQ, JS, SY, AM, KAF, JG, CAA, YS, SRN, MZ, VPP, SC, and SKH annotated the genome. XQ and HJ contributed bioinformatics support. YD, SD-R, CB, HS, GC, GW, DM, LC, and RAG composed the sequencing and finishing team. DAG, BEM, SKH, and GMW assisted in critical review of the manuscript. All authors read and approved the final manuscript.

## Additional data files

The following additional data files are available with the online version of this paper. Additional data file 1 is a list of the ORFs unique to OG1RF compared to V583 with their OG1RF locus tag, location in the genome, and definition. Additional data file 2 is a list of genes encoding proteins with a WxL domain in OG1RF and/or V583. Additional data file 3 shows the results of the mouse peritonitis model using OG1RF and V583, with the statistical analysis. Additional data file 4 is a list of the significant primers used in this study.

## Supplementary Material

Additional data file 1 OG1RF locus tag, location in the genome, and definition are given. Click here for file

Additional data file 2 Genes encoding proteins with a WxL domain in OG1RF and/or V583. Click here for file

Additional data file 3 Results of the mouse peritonitis model using OG1RF and V583, with the statistical analysis. Click here for file

Additional data file 4 The significant primers used in this study. Click here for file
