# Supplementary material for: Large scale variation in Enterococcus faecalis illustrated by the genome analysis of strain OG1RF
Source: Genome Biol. 2008 Jul 8;9(7):R110. doi: 10.1186/gb-2008-9-7-r110 (PMC2530867; doi:10.1186/gb-2008-9-7-r110)
Supplement: Additional data file 1 — OG1RF locus tag, location in the genome, and definition are given. [file gb-2008-9-7-r110-S1.pdf]

# Supplemental Table S1: ORFs unique to OG1RF

| ID[a] | START | STOP | | SIZE | DEFINITION | GI number[b] | Tn*917* [c] |
|---|---|---|---|---|---|---|---|
| OG1RF_0001 | 35502 | 36008 | +[d] | 169 | hypothetical membrane protein | | |
| OG1RF_0002 | 79572 | 80087 | + | 172 | hypothetical protein | | |
| OG1RF_0003 | 80087 | 80143 | + | 19 | hypothetical protein | | |
| OG1RF_0004 | 80128 | 80229 | - | 34 | hypothetical protein | | |
| OG1RF_0005 | 80242 | 80757 | + | 172 | conserved hypothetical protein | 116873726 | |
| OG1RF_0006 | 80757 | 82154 | + | 466 | conserved hypothetical protein | 25012005 | |
| OG1RF_0007 | 82151 | 82456 | + | 102 | hypothetical protein | | |
| OG1RF_0008 | 82472 | 82732 | + | 87 | hypothetical protein | | |
| OG1RF_0009 | 143070 | 143468 | + | 133 | conserved hypothetical membrane protein | 25010299 | |
| OG1RF_0010 | 143468 | 144706 | + | 413 | possible lipase | 25012005 | |
| OG1RF_0011 | 144716 | 145030 | + | 105 | hypothetical protein | | |
| OG1RF_0012 | 145035 | 145301 | + | 89 | hypothetical protein | | |
| OG1RF_0013 | 294095 | 295453 | + | 453 | secreted antigen (EF0394)[e] | 29375030 | |
| OG1RF_0014 | 319060 | 319215 | + | 52 | hypothetical protein | | |
| OG1RF_0015 | 319212 | 320333 | + | 374 | conserved hypothetical protein | 81097058 | |
| OG1RF_0016 | 394987 | 395835 | - | 283 | conserved hypothetical protein | 72162835 | |
| OG1RF_0017 | 416527 | 420540 | + | 1338 | CRISPR associated protein (*cas_csn1* ) | 47097148 | C11 |
| OG1RF_0018 | 420541 | 421407 | + | 289 | CRISPR associated protein (*cas1* ) | 47097147 | |
| OG1RF_0019 | 421404 | 421733 | + | 110 | CRISPR associated protein (*cas2* ) | 15675043 | |
| OG1RF_0020 | 421734 | 422393 | + | 220 | CRISPR associated protein (*cas_csn2* ) | 47097145 | |
| OG1RF_0022 | 423326 | 423682 | + | 119 | hypothetical membrane protein | | |
| OG1RF_0023 | 504238 | 504375 | - | 46 | hypothetical protein | | |
| OG1RF_0024 | 530916 | 531041 | - | 42 | hypothetical protein | | |
| OG1RF_0025 | 725858 | 726289 | + | 144 | conserved hypothetical protein | 29375549 | C10 |
| OG1RF_0026 | 726595 | 726726 | - | 44 | hypothetical protein | | |
| OG1RF_0027 | 726742 | 727125 | + | 128 | hypothetical secreted protein | | |
| OG1RF_0028 | 727219 | 727386 | + | 56 | hypothetical protein | | |
| OG1RF_0029 | 727422 | 727817 | + | 132 | hypothetical protein | | |
| OG1RF_0030 | 727833 | 728126 | + | 98 | hypothetical protein | | |
| OG1RF_0031 | 728205 | 728615 | + | 137 | conserved hypothetical secreted protein | 76787389 | |
| OG1RF_0032 | 728608 | 728901 | + | 98 | hypothetical protein | | |
| OG1RF_0033 | 729061 | 729393 | + | 111 | pseudogene of conserved hypothetical protein | 125717807 | |
| OG1RF_0034 | 729395 | 730957 | + | 521 | conserved hypothetical protein | 77409424 | |
| OG1RF_0035 | 730977 | 731261 | + | 95 | hypothetical protein | | |
| OG1RF_0036 | 731269 | 731394 | + | 42 | hypothetical protein | 29374661 | |
| OG1RF_0037 | 731395 | 731574 | + | 60 | hypothetical protein | | |
| OG1RF_0038 | 798315 | 798425 | + | 37 | hypothetical protein | | |
| OG1RF_0039 | 825385 | 828604 | + | 1073 | probable adhesin (frameshift) | 69244908 | |
| OG1RF_0040 | 828708 | 829034 | + | 109 | hypothetical protein | 29376423 | |
| OG1RF_0041 | 829037 | 829411 | + | 125 | conserved hypothetical protein | 29376422 | A9 |
| OG1RF_0042 | 829411 | 830766 | + | 452 | probable cell division protein FtsK/SpoIIIE | 69244912 | C8 |
| OG1RF_0043 | 830778 | 831782 | + | 335 | DNA (cytosine-5-)-methyltransferase | 69244913 | |
| OG1RF_0044 | 831799 | 832119 | + | 107 | conserved hypothetical protein | 69244914 | |
| OG1RF_0045 | 832358 | 833539 | + | 394 | probable replication initiation protein | 29376415 | |
| OG1RF_0046 | 833557 | 833778 | + | 74 | conserved hypothetical transposon protein | 29376412 | |
| OG1RF_0047 | 833799 | 834299 | + | 167 | antirestriction protein ArdA | 29376411 | |

| ID[a] | START | STOP | | SIZE | DEFINITION | GI number[b] | Tn*917*[c] |
|---|---|---|---|---|---|---|---|
| OG1RF_0048 | 834381 | 835331 | + | 317 | conserved hypothetical protein | 116872488 | A3 |
| OG1RF_0049 | 835333 | 835797 | + | 155 | conserved hypothetical protein | 116872489 | A6 |
| OG1RF_0050 | 835797 | 837683 | + | 629 | possible DNA repair protein RecN | 116872490 | A12 |
| OG1RF_0051 | 837843 | 838136 | + | 98 | possible conjugative transposon membrane protein | 69244919 | |
| OG1RF_0052 | 838114 | 840582 | + | 823 | conserved hypothetical protein | 29376407 | B3 |
| OG1RF_0053 | 840566 | 842386 | + | 607 | conserved hypothetical membrane protein | 69247878 | |
| OG1RF_0054 | 842447 | 843451 | + | 335 | NlpC domain lipoprotein/C40 family peptidase | 16803144 | |
| OG1RF_0055 | 843465 | 844403 | + | 313 | possible conjugative transposon protein | 15923389 | B6 |
| OG1RF_0056 | 844571 | 845689 | + | 373 | possible ATP-binding protein | 27466959 | |
| OG1RF_0057 | 846091 | 846867 | - | 259 | possible HAD family haloacid dehalogenase hydrolase | 27467977 | |
| OG1RF_0058 | 847259 | 847663 | + | 135 | conserved hypothetical protein | 125718781 | |
| OG1RF_0059 | 847698 | 848345 | + | 216 | possible glutathionine S-transferase | 39933897 | |
| OG1RF_0060 | 848702 | 850171 | + | 490 | conserved hypothetical protein | 69245042 | C6 |
| OG1RF_0061 | 850171 | 850506 | + | 112 | hypothetical protein | | |
| OG1RF_0062 | 850525 | 851037 | + | 171 | pseudogene of possible Cna family adhesin | 69244094 | |
| OG1RF_0063 | 851158 | 855372 | + | 1405 | probable collagen adhesion protein | 52140206 | C1 |
| OG1RF_0064 | 855608 | 855853 | + | 82 | hypothetical protein | | |
| OG1RF_0065 | 855884 | 855976 | + | 31 | possible extracellular protein | | |
| OG1RF_0066 | 856019 | 856243 | + | 75 | hypothetical protein | | |
| OG1RF_0067 | 856266 | 857285 | + | 340 | hypothetical protein | 66821251 | |
| OG1RF_0068 | 857430 | 857855 | + | 142 | conserved hypothetical protein | 69247694 | B2 |
| OG1RF_0069 | 857845 | 858081 | + | 79 | conserved hypothetical protein | 69247695 | B7 |
| OG1RF_0070 | 858159 | 858464 | - | 102 | probable transcriptional regulator | 116512576 | |
| OG1RF_0071 | 858566 | 859177 | + | 204 | nitroreductase | 116512577 | |
| OG1RF_0072 | 859418 | 859828 | - | 137 | conserved hypothetical membrane protein | 118587099 | |
| OG1RF_0073 | 859821 | 860273 | - | 151 | possible LytR family response regulator | 81428169 | |
| OG1RF_0074 | 860315 | 860752 | - | 146 | conserved hypothetical membrane protein | 118587101 | |
| OG1RF_0075 | 860794 | 860913 | - | 40 | hypothetical protein | | |
| OG1RF_0076 | 860924 | 861532 | + | 203 | hypothetical membrane protein | | |
| OG1RF_0077 | 861997 | 862107 | + | 37 | hypothetical protein | | |
| OG1RF_0078 | 862178 | 864250 | + | 691 | probable ATP-binding nuclease | 56963170 | |
| OG1RF_0079 | 864243 | 866105 | + | 621 | excision endonuclease subunit UvrD | 28867288 | |
| OG1RF_0080 | 866442 | 866777 | + | 112 | pseudogene of nitroreductase | 24378844 | |
| OG1RF_0081 | 867224 | 867976 | + | 251 | hypothetical protein | | C7 |
| OG1RF_0082 | 868056 | 869654 | + | 533 | hypothetical protein | | |
| OG1RF_0083 | 869700 | 869855 | + | 52 | hypothetical membrane protein | | |
| OG1RF_0084 | 870492 | 871022 | + | 177 | hypothetical protein | | |
| OG1RF_0085 | 871059 | 871832 | + | 258 | hypothetical membrane protein | | |
| OG1RF_0086 | 872173 | 872262 | + | 30 | hypothetical protein | | |
| OG1RF_0087 | 872259 | 872630 | + | 124 | conserved hypothetical protein | 69244684 | |
| OG1RF_0088 | 872681 | 873829 | + | 383 | probable integrase | 69244690 | A8 |
| OG1RF_0089 | 873831 | 874004 | + | 58 | hypothetical protein | | |
| OG1RF_0090 | 1145408 | 1145701 | + | 98 | conserved hypothetical protein | 125624746 | |
| OG1RF_0091 | 1145768 | 1149205 | + | 1146 | conserved hypothetical membrane protein | 116512650 | B11 |
| OG1RF_0092 | 1149208 | 1149705 | + | 166 | hypothetical protein | | |
| OG1RF_0093 | 1149720 | 1149989 | + | 90 | hypothetical protein | | |
| OG1RF_0094 | 1149986 | 1151158 | + | 391 | conserved hypothetical protein | 15896941 | |
| OG1RF_0095 | 1151139 | 1155569 | + | 1477 | cell division protein FtsK/SpoIIIE | 15896940 | |
| OG1RF_0096 | 1155566 | 1155976 | + | 137 | conserved hypothetical protein | 15896939 | |

| ID[a] | START | STOP | | SIZE | DEFINITION | GI number[b] | Tn*917*[c] |
|---|---|---|---|---|---|---|---|
| OG1RF_0097 | 1155977 | 1156231 | + | 85 | hypothetical protein | | |
| OG1RF_0098 | 1156244 | 1156600 | + | 119 | conserved hypothetical protein | 16799474 | D5 |
| OG1RF_0099 | 1156591 | 1158204 | + | 538 | conserved hypothetical protein | 16799475 | |
| OG1RF_0100 | 1158185 | 1158970 | + | 262 | conserved hypothetical protein | 16799476 | |
| OG1RF_0101 | 1159005 | 1159523 | + | 173 | pseudogene of conserved hypothetical protein | 16799475 | |
| OG1RF_0102 | 1159549 | 1160343 | + | 265 | conserved hypothetical protein | 116874092 | |
| OG1RF_0103 | 1160398 | 1160973 | + | 192 | pseudogene of conserved hypothetical protein | 16799475 | |
| OG1RF_0104 | 1161120 | 1161830 | + | 237 | conserved hypothetical protein | 116871754 | |
| OG1RF_0105 | 1162107 | 1164602 | + | 832 | pseudogene of cell division protein FtsK/SpoIIIE | 23098771 | |
| OG1RF_0106 | 1164617 | 1165488 | + | 290.6 | conserved hypothetical membrane protein (frameshift) | 52144636 | |
| OG1RF_0107 | 1165501 | 1165947 | + | 149 | conserved hypothetical protein | | B5 |
| OG1RF_0108 | 1165967 | 1166239 | + | 91 | hypothetical protein | | |
| OG1RF_0109 | 1166253 | 1166897 | + | 215 | hypothetical protein | | |
| OG1RF_0110 | 1166913 | 1168454 | + | 514 | pseudogene of conserved hypothetical protein | 49477502 | |
| OG1RF_0111 | 1168442 | 1169149 | + | 236 | hypothetical protein | | |
| OG1RF_0112 | 1169175 | 1169873 | + | 233 | pseudogene of conserved hypothetical protein | 116871477 | |
| OG1RF_0113 | 1169861 | 1170577 | + | 239 | hypothetical membrane protein | | |
| OG1RF_0114 | 1170835 | 1171263 | + | 143 | conserved hypothetical protein | 116871479 | D1 |
| OG1RF_0115 | 1171272 | 1171721 | + | 150 | conserved hypothetical protein | 49477505 | |
| OG1RF_0116 | 1171795 | 1172295 | + | 167 | hypothetical membrane protein | | |
| OG1RF_0117 | 1272374 | 1272883 | + | 170 | ECF family DNA directed RNA polymerase sigma | 89096444 | |
| OG1RF_0118 | 1273721 | 1273981 | + | 87 | hypothetical protein | 52081095 | |
| OG1RF_0119 | 1272884 | 1273981 | + | 366 | conserved hypothetical protein | 34496255 | |
| OG1RF_0120 | 1290363 | 1291844 | + | 494 | BglG family transcriptional antiterminator | 47095712 | |
| OG1RF_0121 | 1291834 | 1292286 | + | 151 | PTS family porter component IIA | 15901457 | A4 |
| OG1RF_0122 | 1292286 | 1292603 | + | 106 | PTS family porter component IIB | 15901455 | A5 |
| OG1RF_0123 | 1292623 | 1293072 | + | 150 | PTS family porter component IIA | 76788396 | B9 |
| OG1RF_0124 | 1293182 | 1294282 | + | 367 | PTS family porter component IIC | 15901454 | A2 |
| OG1RF_0125 | 1294420 | 1295109 | + | 230 | ribulose-phosphate 3-epimerase | 15901453 | |
| OG1RF_0126 | 1512391 | 1513491 | - | 367 | ABC superfamily ATP binding cassette transporter, membrane protein | 69245312 | |
| OG1RF_0127 | 1513484 | 1514176 | - | 231 | ABC superfamily ATP binding cassette transporter, binding protein | 124520827 | C5 |
| OG1RF_0128 | 1602425 | 1603462 | - | 346 | conserved hypothetical membrane protein | 16799878 | |
| OG1RF_0129 | 1603504 | 1604055 | - | 184 | WxL domain surface protein | 15673181 | |
| OG1RF_0130 | 1604048 | 1604407 | - | 120 | LPxTG-domain surface protein | | |
| OG1RF_0131 | 1604383 | 1607043 | - | 887 | conserved hypothetical protein | 15673179 | |
| OG1RF_0132 | 1607271 | 1608728 | + | 486 | conserved hypothetical protein | 116872203 | |
| OG1RF_0133 | 1608837 | 1610759 | + | 641 | conserved hypothetical membrane protein | 77974891 | |
| OG1RF_0134 | 1610944 | 1611780 | - | 279 | PP-loop superfamily ATP-binding protein | 116493418 | |
| OG1RF_0135 | 1611886 | 1612599 | - | 238 | ABC superfamily ATP binding cassette transporter, ABC protein | 55820385 | |
| OG1RF_0136 | 1612601 | 1613386 | - | 262 | [illegible] protein | 55822275 | |
| OG1RF_0137 | 1613386 | 1614372 | - | 329 | [illegible] protein | 55822274 | |
| OG1RF_0138 | 1614391 | 1615047 | - | 219 | transcriptional regulator | 116493423 | |
| OG1RF_0139 | 1615207 | 1616481 | + | 425 | conserved hypothetical protein | 116493422 | |
| OG1RF_0140 | 1616519 | 1617262 | + | 248 | NCAIR mutase | 116493421 | |
| OG1RF_0141 | 1664191 | 1665858 | - | 556 | glycosyl hydrolase | 81428811 | B1 |
| OG1RF_0142 | 1665873 | 1667582 | - | 570 | PTS family porter component IIABC | 30023990 | C3 |
| OG1RF_0143 | 1667742 | 1668449 | - | 236 | GntR family transcriptional regulator | 82745913 | |
| OG1RF_0144 | 1687265 | 1688902 | - | 546 | conserved hypothetical protein | 15923072 | A10 |

| ID[a] | START | STOP | | SIZE | DEFINITION | GI number[b] | Tn*917*[c] |
|---|---|---|---|---|---|---|---|
| OG1RF_0145 | 1688928 | 1689056 | - | 43 | pseudogene of conserved hypothetical protein | 29376482 | |
| OG1RF_0146 | 1689467 | 1690453 | - | 329 | conserved hypothetical protein | 51245884 | |
| OG1RF_0147 | 1690471 | 1691562 | - | 364 | DNA (cytosine-5-)-methyltransferase | 42779446 | |
| OG1RF_0148 | 1716523 | 1716969 | - | 149 | conserved hypothetical secreted protein | 69244407 | |
| OG1RF_0150 | 1777278 | 1778780 | + | 501 | conserved hypothetical protein | 116873914 | A7 |
| OG1RF_0151 | 1783816 | 1784796 | - | 327 | possible glycosyltransferase | 16804588 | |
| OG1RF_0152 | 1786113 | 1786217 | + | 35 | hypothetical protein | | |
| OG1RF_0153 | 1786477 | 1786602 | - | 42 | hypothetical protein | | |
| OG1RF_0154 | 1787005 | 1787118 | - | 38 | hypothetical protein | | |
| OG1RF_0155 | 1787521 | 1788945 | - | 475 | MOP superfamily multidrug/oligosaccharidyl-lipid/polysaccharide flippase transporter | 124521111 | |
| OG1RF_0156 | 1788993 | 1790378 | - | 462 | probable O-antigen polymerase (EF2169) | 29376677 | |
| OG1RF_0157 | 1790393 | 1791604 | - | 404 | CDP-glycerol:poly(glycerophosphate) glycerophosphotransferase | 106887729 | |
| OG1RF_0158 | 1791650 | 1792051 | - | 134 | glycerol-3-phosphate cytidylyltransferase | 28377193 | |
| OG1RF_0159 | 1792080 | 1793285 | - | 402 | CDP-glycerol:poly(glycerophosphate) glycerophosphotransferase | 106887729 | |
| OG1RF_0160 | 1793311 | 1794291 | - | 327 | glycosyltransferase (EF2170) | 29376678 | |
| OG1RF_0161 | 1794302 | 1795279 | - | 326 | glycosyltransferase | 55823030 | |
| OG1RF_0162 | 1796446 | 1796670 | + | 75 | pseudogene of transposase | 69248334 | |
| OG1RF_0163 | 1796720 | 1799176 | - | 819 | conserved hypothetical protein | 29376682 | C2 |
| OG1RF_0164 | 1799420 | 1799623 | - | 68 | pseudogene of licD-related protein | 29376683 | |
| OG1RF_0165 | 1846772 | 1852468 | - | 1899 | probable adhesin (EF2224) | 29376731 | |
| OG1RF_0166 | 1873057 | 1873362 | - | 102 | PTS family possible cellobiose porter component IIB | 69244697 | |
| OG1RF_0167 | 1873390 | 1874985 | - | 532 | SSS family solute:sodium (Na+) symporter | 56962210 | |
| OG1RF_0168 | 1875009 | 1875899 | - | 297 | myo-inositol catabolism protein IolE | 28210254 | |
| OG1RF_0169 | 1875921 | 1876940 | - | 340 | inositol 2-dehydrogenase | 28210255 | A11 |
| OG1RF_0170 | 1876964 | 1877971 | - | 336 | inositol 2-dehydrogenase | 16081021 | |
| OG1RF_0171 | 1878145 | 1879602 | - | 486 | methylmalonate-semialdehyde dehydrogenase (acylating) | 52082532 | |
| OG1RF_0172 | 1879642 | 1880481 | - | 280 | myo-inositol catabolism protein IolB | 16799479 | D3 |
| OG1RF_0173 | 1880520 | 1882436 | - | 639 | myo-inositol catabolism protein IolD | 15614881 | |
| OG1RF_0174 | 1882447 | 1883481 | - | 345 | 2-dehydro-3-deoxygluconokinase | 18309069 | |
| OG1RF_0175 | 1883639 | 1884655 | - | 339 | DNA binding protein | 15890504 | |
| OG1RF_0176 | 1884841 | 1886676 | - | 612 | membrane GTP-binding protein LepA (EF2352) | 29376853 | |
| OG1RF_0177 | 1914552 | 1914887 | - | 112 | hypothetical protein | | |
| OG1RF_0178 | 1914891 | 1915181 | - | 97 | conserved hypothetical protein | 125718554 | |
| OG1RF_0179 | 1915195 | 1916508 | - | 438 | conserved hypothetical protein | 125718555 | |
| OG1RF_0180 | 1917467 | 1918003 | - | 179 | conserved hypothetical protein | 125718556 | |
| OG1RF_0181 | 1923812 | 1925998 | - | 729 | possible ATP-binding protein | 106894227 | |
| OG1RF_0182 | 1926001 | 1927263 | - | 421 | possible methyltransferase | 42784651 | |
| OG1RF_0183 | 1943002 | 1943193 | - | 64 | hypothetical protein | | |
| OG1RF_0184 | 2000283 | 2004539 | - | 1419 | cell surface anchor protein CnaB (EF2466) | 29376960 | |
| OG1RF_0185 | 2018677 | 2018778 | - | 34 | hypothetical protein | | |
| OG1RF_0186 | 2028700 | 2033007 | - | 1436 | probable adhesin (EF2505) | 29376997 | |
| OG1RF_0187 | 2036086 | 2036412 | + | 109 | possible LIV-E family branched chain amino acid exporter AzlD | 69245522 | |
| OG1RF_0188 | 2200969 | 2202471 | + | 501 | conserved hypothetical membrane protein | 92089170 | |
| OG1RF_0189 | 2216258 | 2217355 | - | 366 | conserved hypothetical membrane protein | 29377266 | |
| OG1RF_0190 | 2273034 | 2274098 | + | 355 | conserved hypothetical lipoprotein | | |
| OG1RF_0191 | 2281411 | 2282121 | - | 237 | M15 family muramoylpentapeptide carboxypeptidase | 29376800 | |
| OG1RF_0192 | 2282219 | 2283331 | - | 371 | sensor histidine kinase VanSG | 119635646 | C4 |
| OG1RF_0193 | 2283324 | 2284028 | - | 235 | response regulator VanRG | 119635645 | B10 |

| ID[a] | START | STOP | | SIZE | DEFINITION | GI number[b] | Tn*917*[c] |
|---|---|---|---|---|---|---|---|
| OG1RF_0194 | 2357843 | 2359177 | - | 445 | probable S1 family extracellular protease | 116873306 | |
| OG1RF_0195 | 2359316 | 2360152 | + | 279 | conserved hypothetical protein | 116515314 | D2 |
| OG1RF_0196 | 2375652 | 2375762 | + | 37 | enterococcal conserved hypothetical protein | | |
| OG1RF_0197 | 2502166 | 2502267 | + | 34 | hypothetical protein | | |
| OG1RF_0198 | 2514398 | 2515117 | - | 240 | response regulator | 47567135 | |
| OG1RF_0199 | 2515119 | 2516447 | - | 443 | sensor histidine kinase | 47567134 | |
| OG1RF_0200 | 2516676 | 2517908 | - | 411 | ABC superfamily ATP binding cassette transporter, membrane protein | 16078054 | |
| OG1RF_0201 | 2517910 | 2518818 | - | 303 | ABC superfamily ATP binding cassette transporter ABC protein | 56965674 | |
| OG1RF_0202 | 2546408 | 2546986 | - | 193 | phosphatidyltransferase | 29377600 | |
| OG1RF_0203 | 2547406 | 2547639 | - | 78 | pseudogene of conserved hypothetical protein | 16799475 | |
| OG1RF_0204 | 2547682 | 2547975 | - | 98 | hypothetical protein | | |
| OG1RF_0205 | 2547985 | 2548284 | - | 100 | hypothetical protein | | |
| OG1RF_0206 | 2548307 | 2549707 | - | 467 | pseudogene of conserved hypothetical protein | 16799475 | |
| OG1RF_0207 | 2549698 | 2550060 | - | 121 | conserved hypothetical protein | 22855014 | |
| OG1RF_0208 | 2550070 | 2550324 | - | 85 | conserved hypothetical protein | 125718209 | A1 |
| OG1RF_0209 | 2554926 | 2555630 | - | 235 | WxL domain surface protein (EF3154, EF3153*)* | 29377607 | C9 |
| OG1RF_0210 | 2555726 | 2556439 | - | 238 | WxL domain surface protein (EF3154, EF3153) | 29377606 | |
| OG1RF_0211 | 2562516 | 2562659 | - | 48 | enterococcal conserved hypothetical protein | 29377690 | |
| OG1RF_0212 | 2563068 | 2563193 | - | 42 | enterococcal conserved hypothetical protein | | |
| OG1RF_0213 | 2589319 | 2593980 | - | 1554 | WxL domain surface protein (EF3188) | 29377638 | |
| OG1RF_0214 | 2615176 | 2617656 | - | 827 | probable choline binding protein | 29377145 | |
| OG1RF_0215 | 2618051 | 2618206 | - | 52 | hypothetical protein | | |
| OG1RF_0216 | 2618199 | 2618339 | - | 47 | hypothetical protein | | |
| OG1RF_0217 | 2618802 | 2619362 | - | 187 | hypothetical protein | | |
| OG1RF_0218 | 2619552 | 2622569 | - | 1006 | probable phosphatidylglycerol--membrane-oligosaccharide glycerophosphotransferase | 69244263 | |
| OG1RF_0219 | 2635203 | 2635340 | - | 46 | enterococcal conserved hypothetical protein | | |
| OG1RF_0220 | 2635352 | 2635693 | - | 114 | probable endoribonuclease MazF | 69244828 | |
| OG1RF_0221 | 2635693 | 2635923 | - | 77 | probable antitoxin MazE | 69244829 | |
| OG1RF_0222 | 2637707 | 2638345 | - | 213 | probable chitin binding protein | 29375001 | |
| OG1RF_0223 | 2641016 | 2641120 | + | 35 | enterococcal conserved hypothetical protein (EF3249) | 29377692 | |
| OG1RF_0224 | 2641315 | 2646006 | - | 1564 | WxL domain surface protein (EF3248) | 29377691 | C12 |
| OG1RF_0225 | 2646355 | 2648313 | - | 653 | WxL domain surface protein (EF3250) | 29377693 | |
| OG1RF_0226 | 2648416 | 2649352 | - | 312 | conserved hypothetical membrane protein | 29377694 | |
| OG1RF_0227 | 2649683 | 2653549 | - | 1289 | WxL domain surface protein (EF3252) | 29377695 | |
| OG1RF_0228 | 1714318 | 1715325 | | 336 | probable DNA methytransferase | 69244404 | |

[a] Only the ORFs unique in OG1RF were labeled

[b] The GI number of the best hit or the most relevent is indicated

[c] New Tn*917* insertion mutants identified in OG1RF unique sequence (plate 7 with coordinates as indicated).

[d] Orientation of the ORF

[e] An "EFxxxx" locus tag number in parentheses indicates polymorphism at the protein level between V583 and OG1RF
