# Supplementary material for: Large scale variation in Enterococcus faecalis illustrated by the genome analysis of strain OG1RF
Source: Genome Biol. 2008 Jul 8;9(7):R110. doi: 10.1186/gb-2008-9-7-r110 (PMC2530867; doi:10.1186/gb-2008-9-7-r110)
Supplement: Additional data file 2 — Genes encoding proteins with a WxL domain in OG1RF and/or V583. [file gb-2008-9-7-r110-S2.pdf]

Locus name	Gene name	aa	Category	Comments
EF0392	EF0392	764	WxL	(Brinster *et al.* , 2007a)
EF0713-4	EF0713	245	WxL	
	EF0714	763	WxL	
EF0750-7	EF0750	112	Cwa	Regulated by the *fsr* system (Bourgogne *et al.* 2006)
	EF0751	243	WxL	
	EF0752	259	WxL	
	EF0753	699	WxL	
	EF0754	259	WxL	
	EF0755	364	DUF916	
	EF0756	149	-	
	EF0757	482	-	
EF1172-6	EF1172	370	-	Possible teichoic acid biosynthesis protein B
	EF1173	248	-	WecB/TagA/CpsF family glycosyl transferase
	EF1174	197	-	Hypothetical protein
	EF1175	132	Cwa	
	EF1176	354	DUF916	
EF1216	EF1216	239	WxL	
EF2248-54	EF2248	1004	WxL	
	EF2249	72	-	
	EF2250	647	WxL	probable internalin
	EF2252	109	Cwa	
	EF2253	349	DUF916	
	EF2254	222	WxL	
EF2682-86	EF2682	341	DUF916	
	EF2683	258	WxL	
	EF2684	260	WxL	
	EF2685	122	Cwa	
	EF2686	614	WxL	probable internalin (Brinster *et al.* 2007a and b)
EF2967-70	EF2967	74	-	
	EF2968	122	Cwa	
	EF2969	377	DUF916	
	EF2970	229	WxL	
EF3074-6	EF3074	1294	WxL	
	EF3075	1426	WxL	
	EF3076	120	Cwa	
EF3153-5	EF3153	238	WxL	78% P to OG1RF210 and 61% P to OG1RF209
	EF3154	236	WxL	80% P to OG1RF210 and 64 %P to OG1RF209
	EF3155	232	WxL	
OG1RF_0209-10	OG1RF_0209	235	WxL	
	OG1RF_0210	238	WxL	
EF3181-8	EF3181	483	-	
	EF3182	161	-	
	EF3183	358	DUF916	
	EF3184	252	WxL	
	EF3185	266	WxL	
	EF3186	244	WxL	
	EF3187	129	Cwa	
	EF3188/OG1RF_213	1554	WxL	82% I/ 88% P
EF3248-53	EF3248/OG1RF_0224	1252	WxL	51% I/ 64% P
	EF3250/OG1RF_0225	645	WxL	56% I/ 68% P
	EF3251/OG1RF_0226	311	DUF916	21% I/ 38% P
	EF3252O/G1RF_0227	1265	WxL	54% I/ 66% P
	EF3253	120	Cwa	100% I
OG1RF_0128-0131	OG1RF_0128	346	DUF916	
	OG1RF_0129	184	WxL	
	OG1RF_0130	120	Cwa	
	OG1RF_0131	887	-	
