# Supplementary material for: Large scale variation in Enterococcus faecalis illustrated by the genome analysis of strain OG1RF
Source: Genome Biol. 2008 Jul 8;9(7):R110. doi: 10.1186/gb-2008-9-7-r110 (PMC2530867; doi:10.1186/gb-2008-9-7-r110)

Table S3- Mouse peritonitis model: results and statistical analysis

Organism	CFU/mouse	Hours after inoculation 0	18	24	27	36	39	48	60	72	96	MTD
OG1RF	1.6E+09	9[a]	0	0	0	0	0	0	0	0	0	18[b]
OG1RF	8.0E+08	9	1	0	0	0	0	0	0	0	0	19
OG1RF	4.0E+08	9	3	2	1	0	0	0	0	0	0	22
OG1RF	2.0E+08	9	3	2	2	1	1	1	1	1	1	21
OG1RF	1.0E+08	9	8	8	8	7	7	7	7	7	7	27

Total mice per group = 9 **OG1RF LD_{50} = 1.34x10^8**

Organism	CFU/mouse	Hours after inoculation 0	18	24	27	36	39	48	60	72	96	MTD
V583	1.0E+09	9	9	1	1	1	1	1	1	1	1	24
V583	5.0E+08	9	9	6	6	3	3	3	1	1	1	38
V583	2.5E+08	9	9	7	6	2	2	2	2	1	1	39
V583	1.3E+08	9	9	9	9	5	5	3	1	1	1	45
V583	6.1E+07	9	9	9	8	6	6	6	1	1	1	57
V583	3.0E+07	9	9	9	9	7	7	7	7	7	7	36

Total mice per group = 9 **V583 LD_{50} = 4.80x10^7**

[a] Total mice alive

[b] Mean time to death (MTD in hours) was calculated by arithmetic mean for all mice within a group that died; surviving mice were not included in this calculation (Kieffer *et al.* , 2003). Analysis of variance indicates that the mean time to death for the mice inoculated with OG1RF and the one inoculated with V583 was significantly different (*P* =0.0058)

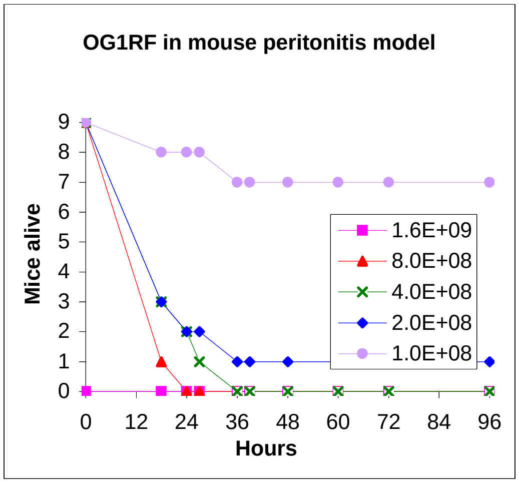

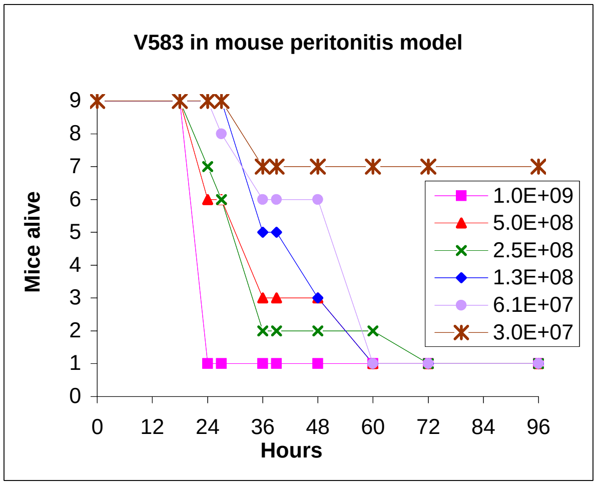

Supplement: Additional data file 3 — Results of the mouse peritonitis model using OG1RF and V583, with the statistical analysis. [file gb-2008-9-7-r110-S3.pdf]
