# Supplementary material for: Large scale variation in Enterococcus faecalis illustrated by the genome analysis of strain OG1RF
Source: Genome Biol. 2008 Jul 8;9(7):R110. doi: 10.1186/gb-2008-9-7-r110 (PMC2530867; doi:10.1186/gb-2008-9-7-r110)
Supplement: Additional data file 4 — The significant primers used in this study. [file gb-2008-9-7-r110-S4.pdf]

Table X: primers used in this study

Primer name	length	Sequence (5' -> 3')
14.8 kb fragment F1	18	gagtgacgattcgacctg
14.8 kb fragment R1	18	ggaacaccaatgacttgc
14.8 kb fragment F2	18	ctgagggcattgaacttg
14.8 kb fragment R2	18	tgattgccgtccctacta
cas_csn2 F	20	ctaccacttgttgcacttca
cas_csn2 R	20	cgattaaagacgttcaaacc
cas1 F	20	tttcattatcggaaatgctt
cas1 R	20	ttgatgttttgttgttggaa
cas_csn1 F	20	gcaaagtcaaccactctctc
cas_csn1 R	20	aagtccaaatcatcaccttg
ef0672-3 jct F	20	tgccaatcatcattgttatc
ef0672-3 jct R	20	cgtgtgaagatgaacgtaga
ef1896 F	18	aagggaccaaccaagaag
ef1896 R	18	tccagattgccaagtagc
iolE F	18	attggacatcaacgcatc
IolE R	18	tgggtgtcgaacctattg
iolR F	18	tccctaatcgccacacta
IolR R	18	ttgctgaaaaagcaggag
